# Overlapping genes in the human and mouse genomes

**DOI:** 10.1186/1471-2164-9-169

**Published:** 2008-04-14

**Authors:** Chaitanya R Sanna, Wen-Hsiung Li, Liqing Zhang

**Affiliations:** 1 Department of Computer Science, Virginia Tech, Blacksburg, USA; 2 Departments of Ecology and Evolution, University of Chicago, Chicago, USA; 3 Program in Genetics, Bioinformatics, and Computational Biology, Virginia Tech, Blacksburg, USA; 4 Research Center for Biodiversity and Genomics Research Center, Academia Sinica, Taiwan

## Abstract

**Background:**

Increasing evidence suggests that overlapping genes are much more common in eukaryotic genomes than previously thought. In this study we identified and characterized the overlapping genes in a set of 13,484 pairs of human-mouse orthologous genes.

**Results:**

About 10% of the genes under study are overlapping genes, the majority of which are different-strand overlaps. The majority of the same-strand overlaps are embedded forms, whereas most different-strand overlaps are not embedded and in the convergent transcription orientation. Most of the same-strand overlapping gene pairs show at least a tenfold difference in length, much larger than the length difference between non-overlapping neighboring gene pairs. The length difference between the two different-strand overlapping genes is less dramatic. Over 27% of the different-strand-overlap relationships are shared between human and mouse, compared to only ~8% conservation for same-strand-overlap relationships. More than 96% of the same-strand and different-strand overlaps that are not shared between human and mouse have both genes located on the same chromosomes in the species that does not show the overlap. We examined the causes of transition between the overlapping and non-overlapping states in the two species and found that 3' UTR change plays an important role in the transition.

**Conclusion:**

Our study contributes to the understanding of the evolutionary transition between overlapping genes and non-overlapping genes and demonstrates the high rates of evolutionary changes in the un-translated regions.

## Background

Overlapping genes are known to be common in viruses, mitochondria, bacteria, and plasmids [1], but are thought to be rare in eukaryotes. This view is changing because recent studies have suggested the existence of many overlapping genes in eukaryotic genomes, including human [2-5], mouse [6], rat [7], fish [8], and flies [9,10].

There are two principal types of overlap: (1) the same-strand overlapping type in which the two genes involved are transcribed from the same strand and (2) the different-strand overlapping type in which the two genes are transcribed from different strands. Most of the recent large-scale analyses in higher eukaryotes have been restricted to different-strand-overlap genes, which are potential sense-antisense gene pairs in which the overlap can affect the regulation of gene expression at the level of transcription, mRNA processing, splicing, or translation [11]. Same-strand-overlap genes have been largely neglected, but a number of such genes have been shown to be functionally important [12,13].

It is important to have a broad comparison between the two types of overlap. In this study we have compiled a list of orthologous gene pairs in human and mouse and identified overlapping genes in both species. We examined the respective frequencies of the two types of overlap and compared the lengths of overlapping regions in the two types. Finally, we studied the evolutionary conservation of overlapping relationships between human and mouse and the possible mechanisms of transition between the overlapping and non-overlapping states.

## Results and Discussion

### Frequencies of different types of overlap

Table 1 shows the statistics of overlapping genes in the human and mouse genomes. Among the 13,484 pairs of orthologous genes, there are 669 (54+615) and 554 (57+497) pairs of overlapping genes in human and mouse, respectively. Because some large genes overlap with multiple genes, the numbers of unique genes involved in overlap in human and mouse are not 2 × 669 = 1338 and 1108 but are only 1219 (1219/13484 = 9.0%) and 1004 (7.4%), respectively. These are likely underestimates of the actual number of overlapping genes because we considered only 13,484 pairs of orthologs in the two genomes. To see whether the frequency of overlapping genes is significantly higher than random expectation, we randomly selected the same number of genes on each chromosome as the observed number, computed the proportion of overlapping genes, and found that only about 0.07% and 0.04% are expected by chance in human and mouse, respectively.

Different-strand overlaps are clearly more common than same-strand overlaps (Table 1); indeed, ~90% of the overlapping pairs are on opposite strands. Interestingly, the pattern is opposite in prokaryotes. A study of overlapping genes in 198 microbial genomes revealed that ~84% of the overlapping genes are on the same strand [1]. There can be multiple reasons for the drastic difference in the prevalence of the two types of overlaps between prokaryotes and eukaryotes. It has been shown that in prokaryotes, same-strand overlaps (aka. tandem overlaps) are mostly in the +1 (2 + 3*n *shared bases) and +2 (1 + 3*n *shared bases) reading frames, whereas different-strand overlaps (aka. antiparallel overlaps) are evenly distributed in the three reading frames [1]. The pattern has been considered to be the product of the intensity of selective pressure on different types of overlaps and the level of independence derived from the plasticity in nucleotide sequence while maintaining a given amino acid sequence [1,14]. Compared to the prokaryotes, most eukaryotes have introns and thus tend to have larger genomes. The more abundant different-strand overlaps in eukaryotes might be a joint effect of more complex gene structures and the role that these overlapping genes play in the regulation of gene expression.

**Table 1:** Summary of the overlapping genes in 13,484 orthologous gene pairs of human and mouse.

Species	Total # of overlapping genes (%)^1^	# of same-strand overlapping pairs (%)	# of different-strand overlapping pairs (%)	# of pairs with overlapping coding regions
Human	1219 (9.0%)	54 (8.1%)	615 (91.9%)	51
Mouse	1004 (7.4%)	57 (10.3%)	497 (89.7%)	28

^1 ^Number of unique genes that are involved in overlap

Based on the relative orientations of the genes involved in overlap, overlapping genes can be further classified into divergent (←→), convergent (→←), and embedded forms (one gene is completely contained in the other) for different-strand overlaps, and embedded and not-embedded forms for same-strand overlaps. Table 2 shows that the majority of same-strand overlaps are embedded forms, accounting for ~89% and ~79% of overlapping genes in the human and mouse genomes, respectively. Different-strand overlaps show a different pattern: in both species, convergent is the most common type (~50%), followed by embedded (~29%) and divergent forms (~21%).

**Table 2:** Classification of overlapping genes based on gene expression orientation.

	Same-strand overlaps		Different-strand overlaps		
	Not-embedded	Embedded	Divergent	Convergent	Embedded

Human	6 (11.1%)	48 (88.9%)	157 (25.5%)	281 (45.7%)	177 (28.8%)
Mouse	12 (21.1%)	45 (78.9%)	80 (16.1%)	268 (53.9%)	149 (30.0%)

We found no previous estimates of the frequencies of different types of same-strand overlaps. On the other hand, there have been some estimates on the proportions of the three types of different-strand overlap. For example, Veeramachaneni et al. (2004) found that ~54% are convergent, ~30% divergent, and ~16% embedded in a sample of 774 different-strand overlaps in human. A similar pattern was observed in mouse for a sample of 542 different-strand overlaps with ~54% convergent, ~37% divergent, and ~9% embedded forms. Shendure and Church [15] reported that ~72% of 185 different-strand-overlap genes in human and mouse are convergent, ~22% are embedded forms, and only ~6% are divergent. Lehner et al. [16] and Yelin et al. [17] also examined the frequencies of different types of different-strand overlaps. A most recent study estimated 25–27% for the convergent, 27–30% for the divergent, and 43–48% for the embedded types of overlap [18]. Therefore, estimates of the frequencies of different types of overlap differed considerably among studies. The differences can be due to a number of reasons such as small sample sizes, different computational methods used to identify sense and antisense genes, different criteria used to filter out spurious different-strand gene pairs, and different experimental data used for the evidence of transcription.

We also examined whether genes involved in overlap share coding regions or not (Table 1). There are no same-strand-overlap genes that share coding regions; therefore, in the same-strand overlaps, one gene resides in another gene's introns. Moreover, there are only 51 genes (51/615 = 8.3%) and 28 genes (28/497 = 5.6%) that involve exon-exon overlaps on opposite strands in human and mouse, respectively. The dearth of genes that share coding regions suggests at least two possibilities. One might be selection against overlap in coding regions. Since there is no selective pressure for a compact genome in eukaryotes (unlike the case of prokaryotes) and since selection on genes sharing coding regions can be strong because overlap may cause interference in transcription, it is preferred that genes do not overlap with each other in coding regions. The second, perhaps the major reason, might be that the majority of overlapping genes evolved from non-overlapping genes, so that they originally had independent coding regions and merging of coding regions rarely occurs. Compared with previous estimates, our estimates of the number of overlapping genes in coding regions are relatively low due to our stringent criterion of requiring that all genes studied here need to be one-to-one orthologs in human and mouse (i.e. the ortholog assignment must have strong phylogenetic support), which eliminates many genes in multi-gene families that have ambiguous ortholog assignments between human and mouse.

### Lengths of overlap regions

**Figure 1:**
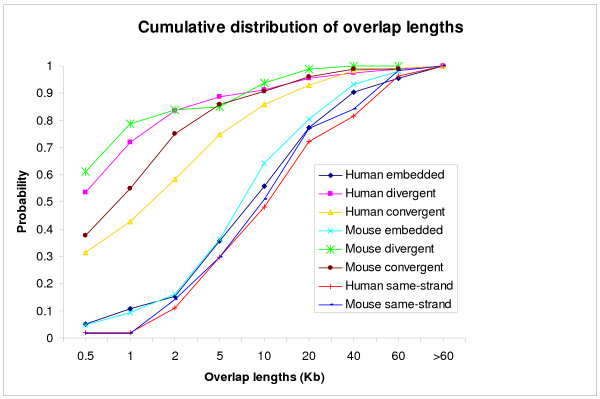
The cumulative distribution of the lengths of overlapping regions. Different – strand overlaps were divided into divergent, convergent, and embedded types.

Figure 1 shows the cumulative distribution of overlap lengths. The two species show very similar patterns. The overlap lengths of the two genes in different-strand overlaps tend to be much shorter than those for same-strand overlaps. For example, ~43% of the overlap regions of different-strand overlaps are shorter than 1 kb, whereas less than 2% of the overlap regions of same-strand overlaps are shorter than 1 kb. For the majority of same-strand overlaps, overlap regions account for 90–100% of the shorter gene but less than 10% of the longer gene, consistent with the observation that the majority of same-strand overlaps are embedded forms (Table 2). For different-strand overlaps, in ~37–46% of the cases the overlap regions account for less than 10% of the length of the shorter gene, and in ~30–34% of the cases the overlap regions account for 90–100% of the length of the shorter gene.

The distribution of lengths of overlap regions seems to reflect the degree of selective constraints on the length of overlap regions. The selective constraint appears to be stronger in different-strand overlaps than in same-strand overlaps. The length of overlap regions and the location of overlap regions in the genes (i.e., 5' UTR, introns, exons, or 3' UTR) may determine the types of expression regulation or correlation between overlapping genes. For example, it has been shown that genes on different strands that overlap in their 5' UTRs tend to have coordinated gene expression [19], whereas genes that overlap in their 3' UTRs seem to be mostly implicated in sense and antisense interaction that causes a negative association between genes' expression [11]. Recently, it has been shown that the lengths of overlap regions can significantly influence the degree of transcriptional interference between *cis *natural antisense genes (*cis*-NATs) – the longer the overlap region, the greater the transcriptional interference in *cis*-NATs [20]. The study of the role of overlap in gene regulation is still in its infancy and much remains to be done to understand how overlapping genes interfere with each other's expression and function.

**Figure 2:**
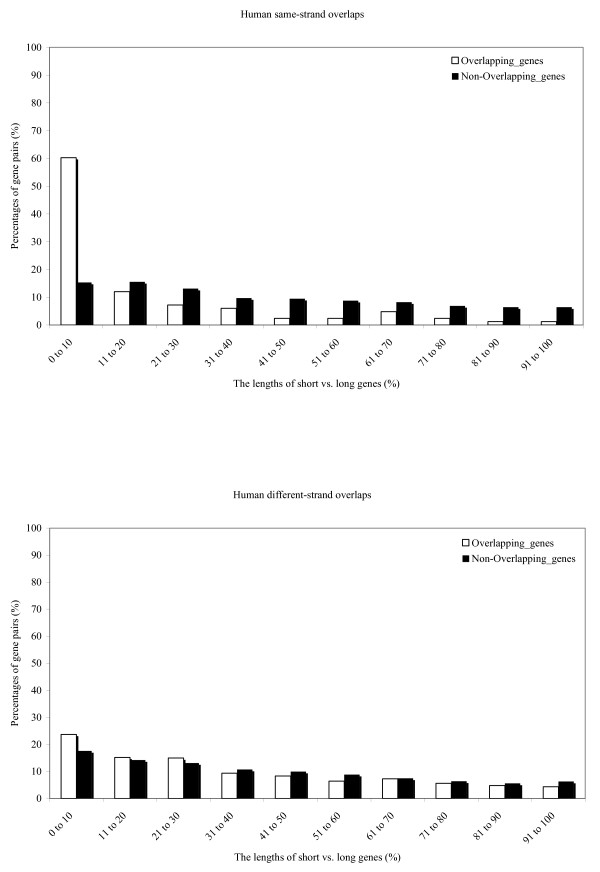
The distributions of the ratios of the lengths of short vs. long genes in overlapping genes (white bars) and in the non-overlapping neighboring gene pairs (black bars). A. Same-strand overlaps. B. Different-strand overlaps.

**Figure 3:**
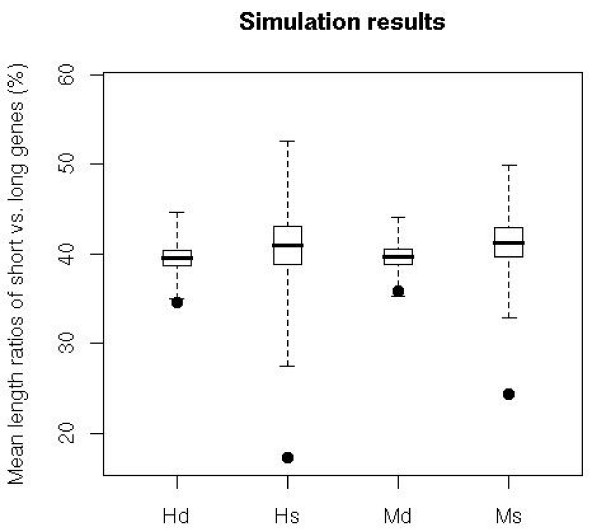
Simulation results on testing the difference between the distributions of length ratios of short vs. long genes for the two types of overlapping genes and the non-overlapping neighboring genes from the 13,484 genes. The X-axis is labeled with the first letter of the species name and the first letter of same- or different-strand (e.g., Hs and Hd refer to same- and different-strand in human respectively). The points are the observed means of length ratios of short vs. long genes. The bar inside each box marks the mean of 10,000 simulations. The whiskers mark the range of mean ratios in 10,000 simulations. The p-values are 0.0006 for mouse different-strand (Md), and the rest are all close to 0.

We calculated the ratios of the lengths of shorter and longer genes for overlapping gene pairs and compared them with the ratios of the lengths of the non-overlapping neighboring genes (shorter vs. longer genes) in the sample of 13,484 genes (Figure 2). The permutation tests show that genes involved in the same-strand overlaps have much lower length ratio than neighboring non-overlapping genes (Figure 3, p-value << 0.001 for both species). This makes intuitive sense because most of the same-strand overlaps are in the form of one gene residing in the other gene's introns. For different-strand overlaps, this restriction on gene lengths appears to be not as pronounced, because the vast majority of different-strand overlaps are not embedded forms. However, in different-strand overlaps the length ratio is still significantly lower than the ratios in non-overlapping neighboring genes (p-values < 0.01).

### Gain and loss of overlapping genes

We examined the shared overlapping relationship between human and mouse. We counted the cases where one gene overlaps with multiple genes as single overlaps. For same-strand overlaps, only 6 pairs of overlapping genes are shared between human and mouse, 31 pairs show overlap in human but not in mouse, and 34 pairs show overlap in mouse but not in human. In contrast, for different-strand overlaps, the proportion of shared overlaps between mouse and human is much higher: 215 pairs are shared between the two species, 335 pairs show overlap in human but not in mouse, and 236 pairs show overlap in mouse but not in human. Therefore, different-strand overlaps are three times more likely to be conserved in the two species than same-strand overlaps (27% vs. 8%). A similar observation was made earlier in a comparison of overlapping genes in human and fugu where ~23.3% of different-strand consecutive relationships were found to be shared between human and fugu, though only 13.5% of the same-strand gene pairs were found to be shared between the two organisms [21].

An interesting question on the evolution of overlapping genes is, "how many overlap gains and losses have occurred in the human and mouse lineages since their divergence"? To address this question, we also examined the overlapping states of the orthologous genes in three other completed mammalian genomes, *Rhesus macaque*, rat, and dog. We required that both genes of an overlapping pair have one-to-one orthologous relationships to the genes in the three additional species, which is a very stringent criterion and reduces greatly the number of gene pairs for our analysis. But it also ensures the confidence in our inference. Using this criterion, we were left with 325 pairs for this analysis (Table 3). We found that there have been 15 and 18 gains of same-strand overlap in the human and mouse lineage, respectively. For different-strand overlaps, there have been 154 gains of overlap in human, of which 50 occurred in the ancestral lineage of human and macaque, 121 gains of overlap in mouse, of which 20 gains occurred in the ancestral lineage of mouse and rat, 13 losses of overlap in mouse, of which 10 occurred in the ancestral lineage of mouse and rat, and 4 losses of overlap in human, of which 3 occurred in the ancestral lineage of human and macaque.

**Table 3:** Overlapping states in orthologous genes of different species.

Species	Same-strand^1^				Different-strand^1^												
human	Y	Y	N		Y	Y	Y	Y	Y	Y	Y	N	N	N	N	N	N
macaque	N	N	N		N	Y	Y	N	N	Y	Y	N	N	N	Y	N	Y
mouse	N	N	Y		N	N	N	N	N	N	N	Y	Y	Y	Y	Y	Y
rat	N	N	N		N	N	N	N	Y	Y	Y	N	Y	N	N	Y	Y
Dog	N	Y	N		N	N	Y	Y	N	N	Y	N	N	Y	N	Y	N
Count	14	1	18		95	50	10	5	4	1	2	95	20	4	2	3	1

^1 ^Y: two genes overlap; N: two genes do not overlap.

Caution must be taken in interpreting the results. Although we took steps in minimizing impact of annotation errors on the analysis, we can not guarantee absolute accuracy. It is well-known that the annotation of UTR is difficult, especially with 5' UTRs. Therefore, it is likely that some of the gains and losses of overlaps seen here are actually annotation artifact. In addition to the annotation problem, incomplete sequencing can also contribute to some of the non-conserved overlaps.

### Mechanisms of the nonoverlapping-overlapping transition

The observation that less than 30% of the overlaps are shared between human and mouse suggests fast gain and/or loss of overlapping genes. However, could the low extent of shared overlapping relationships between species be in part due to inaccurate ortholog assignment? Imagine, for instance, gene *A *and gene *B *overlap in human, gene *A *is correctly assigned to its mouse ortholog gene *A***'**, whereas gene *B *is mistakenly assigned to mouse gene *C*. The observation that gene A and gene C do not overlap in mouse can mislead us to conclude that the overlap is not conserved between species. Therefore, it is important to make sure that the two genes involved in overlap in one species both have correct ortholog assignment in the other species. Since we only used the ortholog prediction in Ensembl, it is likely that some of them are wrong. We therefore cross-validated the Ensembl human-mouse ortholog prediction using 6 additional ortholog prediction databases including HGNC [22], Homologene at NCBI [23], Inparanoid [24], MGI [25], PhIGs [26], and Treefam [27]. These databases, together with Ensembl, have independent methods of ortholog prediction and rely on information extracted from either sequences, phylogenetic trees, chromosomal synteny, or some combinations of these aspects. Therefore, the more databases predict an ortholog assignment, the higher confidence we have for the assignment. We found that in 163 of the 636 pairs that show non-conserved overlap relationships, the ortholog assignments of one or both genes in a pair have no other database support except Ensembl; the remaining 473 pairs have both genes whose ortholog assignments have multiple database support; in fact, the majority of the 473 pairs have more than four databases predicting the same ortholog assignment (see supplementary materials). Therefore, the majority of our ortholog assignments are likely accurate and incorrect ortholog assignment likely contributes little to the low extent of conserved overlaps between human and mouse.

Then what is the cause for the low extent of shared overlapping relationships between human and mouse? In other words, what could be the mechanisms for the fast evolutionary switches between overlapping and non-overlapping genes? One likely mechanism for the switch between overlapping and non-overlapping states is genome rearrangement. It might explain the situation where the two genes under study overlap in one species but are on different chromosomes in the other species. For example, 2 mouse pairs that are orthologous to the human same-strand overlaps are located on different chromosomes and 19 mouse pairs that are orthologous to the human different-strand overlaps are located on different chromosomes. However, these genes amount to less than 4% of the non-conserved gene pairs. Therefore, genome rearrangement is not a major cause for the transition.

**Figure 4:**
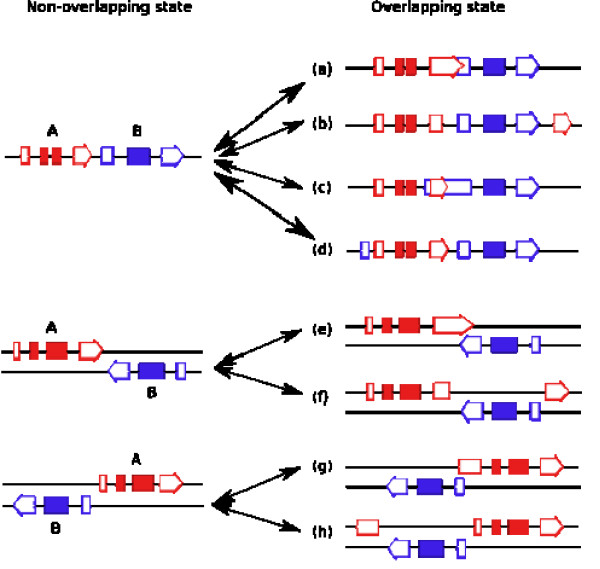
Different scenarios for how two genes A and B may switch from non-overlapping to overlapping, and vice versa. The solid boxes, empty boxes, and empty boxes with arrows are coding exons, 5' UTRs, and 3' UTRs, respectively. Genes A and B are in red and blue, respectively. Cases a-d denote same-strand overlapping genes, while cases e-h denote different-strand overlapping genes. From a non-overlapping state to an overlapping state: (a) Extension of the 3' UTR in gene A due to loss of the transcription stop signal results in extension of gene A into gene B. Here the extension creates only a partial overlap, but a large extension may cover the entire gene B. (b) Emergence of a new 3' UTR exon in gene A results in the complete embedment of gene B within gene A. Unlike case (a), case (b) requires the creation of a new exon, so that it is less likely to occur than case (a). (c) Emergence of a new transcription start site extends the 5' UTR in gene B to partially cover the 3' UTR of gene A. If the extension is long, it may entirely cover gene A. (d) Emergence of a new 5' UTR exon in gene B upstream of gene A results in the embedment of gene A completely inside gene B. (e) Extension of the 3' UTR in gene A due to loss of the transcription stop signal results in extension of gene A into gene B. Here the extension creates only a partial overlap, but a large extension may cover the entire gene B. (f) Emergence of a new 3' UTR exon in gene A results in the complete embedment of gene B inside gene A. (g) Extension of the 5' UTR of gene A in the 5' direction creates a partial overlap of gene A with gene B. (h) Emergence of a new 5' UTR exon in gene A results in the complete embedment of gene B inside gene A. The opposite scenarios to the above would create a transition from the overlapping state to a non-overlapping state of two neighboring genes.

A more likely mechanism for the switch is UTR change, because the majority of the non-conserved overlapping cases are such that the genes that overlap in only one species are located on the same chromosome in the other species. As indicated in Figure 4, for two genes that are neighboring on the same chromosome, the transition between overlapping and non-overlapping states can be simply a matter of gain or loss of either 5' UTR, 3' UTR, or both. For example, a gain of convergent different-strand-overlap genes is fairly simple. The disappearance of a transcription termination site will elongate the RNA transcript and the transcription may run into the 3' of the neighboring gene, so that the two genes become overlap in the 3' end. This may also apply to the case of parallel same-strand neighboring genes, that is, the elongated transcription of an upstream gene may run into the 5' end of the neighboring gene. On the other hand, a gain of divergent different-strand-overlap genes can occur when the transcription start site (TSS) of a gene moves in the 5' direction, so that it includes the TSS of the neighboring gene. Similarly, the loss of convergent different-strand overlaps is simple, namely, the move of a transcription stop site in the 5' direction will shorten the transcription and the overlap may thus disappear. The same comment applies to the case of a parallel overlap.

Given that UTR changes seem to be the major mechanism for the evolutionary switch, we asked the question: what are the relative contributions of 5' change and 3' change? Here we were able to computationally determine whether the switch between overlapping and non-overlapping states is due to 5' change or 3' change by taking advantage of the orthologous gene set and comparing the chromosomal arrangements between overlaps and non-overlaps in human and mouse (see Materials and Methods for details). For the same-strand overlaps, the majority of transitions (~64%) were due to 3' end change and the remaining due to 5' end change (Table 4); the chi-square goodness-of-fit test for equal proportions of 5' and 3' changes is marginally significant (χ^2 ^= 3.27, p-value = 0.07). For the different-strand overlaps, the majority of transitions (~60%) were due to 3' end change; the chi-square goodness-of-fit test for equal proportions of 5' and 3' changes is highly significant (χ^2 ^= 20.93, p-value = 4.8e-6).

Several previous studies examined the transition between overlapping and non-overlapping genes in eukaryotes. For example, Shintani et al. [28] studied the evolutionary origin of TCP1 and ACAT2 that overlap in their 3' UTR on different strands and suggested that the overlap arose during the transitions from therapsid reptiles to mammals and has been retained for >200 million years. They proposed that the two genes were brought together and became overlapping during a chromosome rearrangement. Dan et al. [29] examined the origin of the overlapping genes of *Mink *and *Chrne *and found that the two genes overlap in some mammals but not others owing to different usages of alternative polyadenylation sites.

**Table 4:** Causes of transition between non-overlapping and overlapping states.

Types of overlap	5' change^1^	3' change^2^	Chi-square goodness-of-fit test^3^
Same-strand	16 (36%)	28 (64%)	χ^2 ^= 3.27 (*p *= 0.07)
Different-strand	220 (40%)	327 (60%)	χ^2 ^= 20.93 (*p *= 4.8e-6)

^1 ^The number of cases that show a 5' change. Corresponding percentages are in parenthesis.

^2 ^The number of cases that show a 3' change.

^3 ^Test for equal proportions of 5' and 3' change.

Besides these studies that examined individual cases of overlapping genes in eukaryotes, there have not been any systematic investigations of the causes for the transitions between overlapping and non-overlapping genes, especially for same-strand overlaps. Here, our results on ~600 pairs of overlapping genes in the two mammalian genomes indicate that 3' UTR evolution (gains or losses of 3' transcriptional stop signals) plays a major role in the transition. This seems to be consistent with the recent finding that at least half of all human genes encode multiple transcripts with alternative 3' termini [30]. The higher frequency of transition contributed by 3' UTR changes than by 5' UTR changes implies that it is easier to capture and utilize a downstream alternative termination signal than an upstream alternative start signal. We can get a rough estimate on the respective rates of the two UTRs' evolution. Under the assumption of 80 million years for the divergence time between human and mouse, and the observations that among the total of 857 overlapping gene pairs, there are at least 355 pairs that have gain or loss of 3' UTR and 236 pairs that have gain or loss of 5' UTRs, we estimate that the transition due to 3' UTR change occurs at a rate of ~2.6 per gene pair per billion years and that due to 5' UTR change occurs at a rate of ~1.7 per gene pair per billion years. Thus, rates of gain or loss of UTRs are only somewhat lower than rates of point mutation.

These estimates should be taken with caution as they are crude; although they tend to be conservative, their accuracy can be only as good as the annotation quality of UTRs. Another word of caution is that the higher rate of 3' UTR evolution than 5' UTR could also be partially due to more accurate 3' UTR annotations. Since annotation of UTRs relies to a large extent on ESTs (expressed sequence tags) and since ESTs are biased towards the 3'end of a gene, it is expected that 3' UTRs are better annotated than 5' UTR; thus, alternative usage of 3' UTRs in genes will be more frequently recognized than that of 5' UTRs.

## Conclusion

When gene structure becomes better annotated, we might discover that many genes have multiple alternative 5' or 3' UTRs for transcription, so that two neighboring genes may become overlapping with each other in certain transcribed forms. Thus, it is likely that overlapping genes are even more common than estimated in this study.

## Methods

### Compilation and classification of overlapping genes

We obtained the dataset from Ensembl (version 44) using the data mining tool Biomart. We limited our analysis to protein-coding genes. We did not consider alternative splicing forms of a gene to be overlapping genes (broadly speaking, they may be considered as a special kind of overlapping genes). Because we are especially interested in addressing the evolution of overlapping genes, we require that all genes in our study have strict orthologs between human and mouse genomes. Ensembl contains all-against-all BLASTP results for these two species and the pairwise species comparisons. A gene was added to the list of putative orthologous genes only if the gene in human has a unique best reciprocal hit to a gene in mouse and the ortholog relationship is also supported by a phylogenetic analysis [31]. With these criteria, we identified 13,484 pairs of orthologous genes. These genes were then checked for possible overlaps using the Ensembl gene annotation. According to the annotated start and end positions on chromosomes, two genes were considered to overlap with each other if they share a region longer than 50 base pairs. The overlapping gene pairs were then classified into (1) the same-strand overlap type if the genes involved were on the same strand or (2) the different-strand overlap type if the genes were on opposite strands.

### Length ratio computation

The length comparison was done by computing the ratio of lengths of the shorter and the longer genes in the overlapping gene pair. We considered the same- and different-strand overlaps separately. In addition, we computed the length ratios of the shorter and the longer genes of non-overlapping neighboring gene pairs in the 13,484 genes under study. Here a non-overlapping neighboring gene pair refers to, in the collection of the 13,484 genes, a pair of genes that do not overlap but are next to each other on the same chromosome. In human, there are a total of 4831 such non-overlapping neighboring gene pairs located on the same strand and 4950 pairs on opposite strands. In mouse, there are 4742 such non-overlapping neighboring pairs on the same strand and 4864 pairs on opposite strands. A simple permutation test was performed to formally compare the length distributions between different types of genes. Specifically, for each sample, we randomly picked length ratios with replacement from the length ratios of the genome until the sample contained the same number of length ratios as the one under study. We repeated this procedure 10,000 times to create 10,000 samples. The mean of the length ratios in each sample was calculated and used as the metric for the test. The significance level of the test for same- or different-strand overlaps was determined as the frequency of observing a mean less than the observed mean of length ratios for the respective type of overlap. Note that the Wilcoxon rank sum tests show that the distribution of the lengths of the 13,484 genes is not significantly different from the distribution of the lengths of the overlapping genes (p-values > 0.3), which satisfies the requirement of the permutation tests.

### Models of transition between non-overlapping genes and overlapping genes

Theoretically, two neighboring genes can switch from non-overlapping to overlapping through a 5' change (e.g., gaining a new upstream start signal), a 3' change (e.g., gaining a new downstream termination signal), or a combination of both mechanisms (Fig. 1). Similarly, one can also imagine a switch from overlapping to non-overlapping by a change in the 5' UTR, in the 3' UTR, or a combination of both.

However, it may be difficult to determine computationally whether the transition is due to a 5' or a 3' change alone or a combination of 5' and 3' changes. Fortunately, we can take advantage of the fact that most of the overlapping genes on the same strand are embedded forms and can decide for these cases whether the transition was caused by a 5' or a 3' change using the idea illustrated in cases b and d of Figure 4. Specifically, we compared the order of start positions of a gene pair of embedded same-strand overlaps with those of their orthologous gene pairs in the other species (the dog and rat genomes were also used to reduce annotation errors) that do not overlap but are on the same strand of the chromosome. If the orders of the start positions are the same in the two species, the cause of transition between overlapping and non-overlapping states was due to 3' change (case b); otherwise, it was 5' change (case d). For different-strand overlaps, if the two genes are in convergent orientation, transition between non-overlapping and overlapping states can be due to 3' change in one or both genes (cases e and f). If the two genes are in divergent orientation, the transition can be due to 5' change in one or both genes (cases g and h).

### Gain and loss of overlapping genes

An interesting issue is the gain and loss of overlapping states. To address this question, we also examined the overlapping states of the orthologous genes in three other completed mammalian genomes, *Rhesus macaque*, rat, and dog. We determined gain and loss of overlaps using the maximum parsimony principle [32] and the species tree. We limited this analysis to gene pairs for which both genes have one-to-one orthologous relationships to the genes in the three additional species. The Ensembl ortholog assignment is based on the comparison between the gene tree and the species tree and is thus expected to be more accurate than the assignment of simply reciprocal best hits. Specifically, the one-to-one orthology refers to the case where a gene tree is consistent with the species tree and there is no duplication in the interested species after speciation [31]. This criterion of requiring a one-to-one orthologous relationship in all the additional species is very stringent and reduces greatly the number of gene pairs for our analysis. But it also ensures the confidence in our inference.

## Authors' contributions

CRS performed the analyses and wrote the paper, WHL wrote the paper, and LZ performed the analyses and wrote the paper.

## References

[B1] Johnson ZI, Chisholm SW (2004). Properties of overlapping genes are conserved across microbial genomes. Genome Research.

[B2] Kennerson ML, Nassif NT, Dawkins JL, DeKroon RM, Yang JG, Nicholson GA (1997). The Charcot-Marie-Tooth binary repeat contains a gene transcribed from the opposite strand of a partially duplicated region of the COX10 gene. Genomics.

[B3] Bristow J, Tee MK, Gitelman SE, Mellon SH, Miller WL (1993). Tenascin-X - a Novel Extracellular-Matrix Protein Encoded by the Human Xb Gene Overlapping P450c21b. Journal of Cell Biology.

[B4] Cooper PR, Smilinich NJ, Day CD, Nowak NJ, Reid LH, Pearsall RS, Reece M, Prawitt D, Landers J, Housman DE, Winterpacht A, Zabel BU, Pelletier J, Weissman BE, Shows TB, Higgins MJ (1998). Divergently transcribed overlapping genes expressed in liver and kidney and located in the 11p15.5 imprinted domain. Genomics.

[B5] Veeramachaneni V, Makalowski W, Galdzicki M, Sood R, Makalowska I (2004). Mammalian overlapping genes: The comparative perspective. Genome Research.

[B6] Batshake B, Sundelin J (1996). The mouse genes for the EP(1) prostanoid receptor and the PKN protein kinase overlap. Biochemical and Biophysical Research Communications.

[B7] Adelman JP, Bond CT, Douglass J, Herbert E (1987). 2 Mammalian Genes Transcribed from Opposite Strands of the Same DNA Locus. Science.

[B8] Makalowska I, Lin CF, Makalowski W (2005). Overlapping genes in vertebrate genomes. Computational Biology and Chemistry.

[B9] Misener SR, Walker VK (2000). Extraordinarily high density of unrelated genes showing overlapping and intraintronic transcription units. Biochimica Et Biophysica Acta-Gene Structure and Expression.

[B10] Spencer CA, Gietz RD, Hodgetts RB (1986). Overlapping Transcription Units in the Dopa Decarboxylase Region of Drosophila. Nature.

[B11] Boi S, Solda G, Tenchini ML (2004). Shedding light on the dark side of the genome: Overlapping genes in higher eukaryotes. Current Genomics.

[B12] Cawthon RM, Andersen LB, Buchberg AM, Xu GF, Oconnell P, Viskochil D, Weiss RB, Wallace MR, Marchuk DA, Culver M, Stevens J, Jenkins NA, Copeland NG, Collins FS, White R (1991). Cdna Sequence and Genomic Structure of Ev12b, a Gene Lying within an Intron of the Neurofibromatosis Type-1 Gene. Genomics.

[B13] Williams BAP, Slamovits CH, Patron NJ, Fast NM, Keeling PJ (2005). A high frequency of overlapping gene expression in compacted eukaryotic genomes. Proceedings of the National Academy of Sciences of the United States of America.

[B14] Krakauer DC (2000). Stability and evolution of overlapping genes. Evolution Int J Org Evolution.

[B15] Shendure J, Church GM (2002). Computational discovery of sense-antisense transcription in the human and mouse genomes. Genome Biology.

[B16] Lehner B, Willams G, Campbell RD, Sanderson CM (2002). Antisense transcripts in the human genome. Trends in Genetics.

[B17] Yelin R, Dahary D, Sorek R, Levanon EY, Goldstein O, Shoshan A, Diber A, Biton S, Tamir Y, Khosravi R, Nemzer S, Pinner E, Walach S, Bernstein J, Savitsky K, Rotman G (2003). Widespread occurrence of antisense transcription in the human genome. Nature Biotechnology.

[B18] Galante PA, Vidal DO, de Souza JE, Camargo AA, de Souza SJ (2007). Sense-antisense pairs in mammals: functional and evolutionary considerations. Genome biology.

[B19] Trinklein ND, Aldred SF, Hartman SJ, Schroeder DI, Otillar RP, Myers RM (2004). An abundance of bidirectional promoters in the human genome. Genome Res.

[B20] Osato N, Suzuki Y, Ikeo K, Gojobori T (2007). Transcriptional Interferences in cis Natural Antisense Transcripts of Humans and Mice. Genetics.

[B21] Dahary D, Elroy-Stein O, Sorek R (2005). Naturally occurring antisense: Transcriptional leakage or real overlap?. Genome Research.

[B22] Eyre TA, Ducluzeau F, Sneddon TP, Povey S, Bruford EA, Lush MJ (2006). The HUGO Gene Nomenclature Database, 2006 updates. Nucleic Acids Res.

[B23] Wheeler DL, Barrett T, Benson DA, Bryant SH, Canese K, Chetvernin V, Church DM, DiCuccio M, Edgar R, Federhen S, Geer LY, Helmberg W, Kapustin Y, Kenton DL, Khovayko O, Lipman DJ, Madden TL, Maglott DR, Ostell J, Pruitt KD, Schuler GD, Schriml LM, Sequeira E, Sherry ST, Sirotkin K, Souvorov A, Starchenko G, Suzek TO, Tatusov R, Tatusova TA, Wagner L, Yaschenko E (2006). Database resources of the National Center for Biotechnology Information. Nucleic acids research.

[B24] O'Brien KP, Remm M, Sonnhammer EL (2005). Inparanoid: a comprehensive database of eukaryotic orthologs. Nucleic Acids Res.

[B25] Eppig JT, Bult CJ, Kadin JA, Richardson JE, Blake JA, Anagnostopoulos A, Baldarelli RM, Baya M, Beal JS, Bello SM, Boddy WJ, Bradt DW, Burkart DL, Butler NE, Campbell J, Cassell MA, Corbani LE, Cousins SL, Dahmen DJ, Dene H, Diehl AD, Drabkin HJ, Frazer KS, Frost P, Glass LH, Goldsmith CW, Grant PL, Lennon-Pierce M, Lewis J, Lu I, Maltais LJ (2005). The Mouse Genome Database (MGD): from genes to mice--a community resource for mouse biology. Nucleic acids research.

[B26] Dehal PS, Boore JL (2006). A phylogenomic gene cluster resource: the Phylogenetically Inferred Groups (PhIGs) database. BMC Bioinformatics.

[B27] Li H, Coghlan A, Ruan J, Coin LJ, Heriche JK, Osmotherly L, Li R, Liu T, Zhang Z, Bolund L, Wong GK, Zheng W, Dehal P, Wang J, Durbin R (2006). TreeFam: a curated database of phylogenetic trees of animal gene families. Nucleic Acids Res.

[B28] Shintani S, O'hUigin C, Toyosawa S, Michalova V, Klein J (1999). Origin of gene overlap: The case of TCP1 and ACAT2. Genetics.

[B29] Dan I, Watanabe NM, Kajikawa E, Ishida T, Pandey A, Kusumi A (2002). Overlapping of MINK and CHRNE gene loci in the course of mammalian evolution. Nucleic Acids Research.

[B30] Iseli C, Stevenson BJ, de Souza SJ, Samaia HB, Camargo AA, Buetow KH, Strausberg RL, Simpson AJG, Bucher P, Jongeneel CV (2002). Long-range heterogeneity at the 3 ' ends of human mRNAs. Genome Research.

[B31] Dufayard JF, Duret L, Penel S, Gouy M, Rechenmann F, Perriere G (2005). Tree pattern matching in phylogenetic trees: automatic search for orthologs or paralogs in homologous gene sequence databases. Bioinformatics.

[B32] Li WH (1997). Molecular evolution.

